# Editorial: Molecular Mechanisms and New Therapeutic Targets in Epithelial to Mesenchymal Transition (EMT) and Fibrosis

**DOI:** 10.3389/fphar.2019.01556

**Published:** 2020-01-16

**Authors:** Cecilia Battistelli, Marc Diederich, Timothy Joseph Keane, Pilar Sandoval, Sergio Valente, Raffaele Strippoli

**Affiliations:** ^1^Department of Molecular Medicine, Sapienza University of Rome, Rome, Italy; ^2^Department of Pharmacy, Seoul National University, Seoul, South Korea; ^3^Department of Materials, Imperial College London, London, United Kingdom; ^4^Severo Ochoa Molecular Biology Center (CSIC-UAM), Madrid, Spain; ^5^Department of Chemistry and Technologies of Drugs, Sapienza University of Rome, Rome, Italy; ^6^National Institute for Infectious Diseases L. Spallanzani IRCCS, Rome, Italy

**Keywords:** natural compound, EMT, fibrosis, epigenetics, non-coding RNAs, therapeutic targets

Although the term “epithelial to mesenchymal transformation” was used for the first time by Betty Hay in 1968, the earliest description of the EMT process probably dates back to drawings made by the Nobel Prize Santiago Ramòn y Cajal around 1890 (López-Novoa and Nieto, 2009).

After decades of studies, EMT is now considered a key physiopathological mechanism active in embryogenesis, in fibrotic diseases and in cancer. The scope of this research topic was to provide an updated overview of EMT processes and new therapeutic strategies aimed to target EMT processes. In this Topic, Fintha et al. provided a timely survey on the “fibroblast conversion” hypothesis (with a focus on renal fibrosis), where epithelial cell transdifferentiation plays a crucial role in the generation of myofibroblasts, the main cells implicated in organ fibrosis. The ‘fibroblast conversion' hypothesis has been intensely debated over years, with some contradicting evidence in different experimental systems used.

The contribution of different molecular mechanisms to the initiation of EMT is elucidated by various research articles in this topic. TGFβ is universally known as the main extracellular biochemical EMT promoter. In this topic, the role of other extracellular mediators is discussed. The importance of the gremlin-VEGF2 axis in the genesis of renal fibrosis is discussed by Marquez-Esposito et al. Sonic Hedgehog (SHH) is an extracellular mediator with a role in cellular plasticity in various organs. Magistri et al. analyzed the effect of the inhibition of Smoothened (SMO), an intracellular mediator of SHH signaling, in the control of EMT and invasion in colon carcinoma. Besharat et al. analyzed the intracellular regulation of SHH and interference with VEGF-A/neuropilin signalling in EMT in medulloblastoma. In another study by Rossi et al., the EMT state of different prostate carcinoma cell lines was found differentially responsive to the combination of estrogens and somatostatin derivatives.

Biochemical extracellular mediators promote changes in cell plasticity by inducing specific intracellular pathways. In this topic, the role of pathways alternative to the TGFβ classical pathways, such as SMADs, were analyzed. In particular, using a specific pharmacological inhibitor, Keenan et al. studied the role of casein kinase 1 delta and epsilon in pulmonary fibrosis. In a detailed review article, Stradiot et al. discussed the role of P311, an RNA binding protein implicated in the control of TGFβ translation in organ fibrosis. Moreover, Song et al. analyzed the role of glutamyl-prolyl-tRNA synthetase in the genesis of idiopathic pulmonary fibrosis. Liu et al. analyzed the role of two genes implicated in the oxidative response, PHD1 and KEAP, in controlling bleomycin -induced liver fibrosis in mice.

The changes of the cell proteome occuring during EMT are controlled by a limited number of master genes. Bisceglia et al. explored the mechanism by which the activity of HNF1, a master gene of epithelial differentiation, is impaired by TGFβ via altered recruitment of CBP-p300 acetyltranferase.

Recent studies unveiled the role of epigenetics in the control of EMT/MET dynamics and in general in cellular plasticity. A review article by Carafa et al. analyzed the dual role of sirtuins, class III histone deacetylases, in both promoting and limiting three main processes related to tumor transformation, i.e., EMT, invasion, and metastasis. Besides histone acetyltranferases and deacetylases, epigenetic control is exerted by non-coding RNAs. Among them, microRNAs may post-transcriptionally target the expression of genes relevant for cell plasticity. Jiang et al. analyzed the role of miR-181a in the downregulation of TLR4. TLR mediated activation of NF-κB p65 and ROS pathways controls LPS-induced lung inflammation, which is connected to EMT and fibrosis. Also, Yang et al. demonstrated that miR-374 regulates the inflammatory response in diabetic nephropathy targeting CCL2/MCP1 expression. The induction of this chemokine contributes in turn to increased expression of inflammatory cytokines, such as TNFα and IL-6. Moreover, Besharat et al. analyzed the role of miR-466f-3p in the inhibition of EMT features in medulloblastoma.

Tightly connected with the analysis of molecular mechanisms is the search of new therapeutic targets to control and revert EMT and fibrosis. Emphasis has been given in this topic to natural compounds. Avila-Carrasco et al. provided an extensive survey of natural plant compounds reported to be active in targeting various pathways active in EMT. Other studies analyzed the effect of single derivatives. Wedelolactone, a major coumarin derivative of *E. prostrata*, was reported by Yang et al. to attenuate pulmonary fibrosis in a murine model by acting on AMPK and MAPK pathways. Also, fisetin, a natural flavonoid, was shown by Li et al. to reverse EMT features in triple negative breast cancer cell lines through regulation of (PI3K)-Akt-GSK-3β signaling pathway. Lastly, Xu et al. demonstrated that Tacrolimus, a calcineurin inhibitor, blocks TNF-α secretion and inflammation by interacting with NF-κB p65 activation in cultured epithelial tissue.

In summary, the broad coverage in this Topic provides several new perspectives on molecular aspects and novel therapeutic approaches aimed at counteracting and reversing EMT and fibrosis.

## Author Contributions

All the authors have contributed to the composition and the revision of this Editorial Article.

## Conflict of Interest

The authors declare that the research was conducted in the absence of any commercial or financial relationships that could be construed as a potential conflict of interest.
